# A Four-Item Questionnaire to Measure Problematic Social Media Use: The Social Media Disorder Test

**DOI:** 10.3390/bs13120980

**Published:** 2023-11-28

**Authors:** Lutz Wartberg, Carolin Spindler, Sophia Berber, Katrin Potzel, Rudolf Kammerl

**Affiliations:** 1 Department of Psychology, Faculty of Human Sciences, MSH Medical School Hamburg, 20457 Hamburg, Germany; 2 Department of Education, Chair for Pedagogy with a Focus on Media Education, Friedrich-Alexander-University Erlangen-Nuremberg, 90478 Nuremberg, Germany

**Keywords:** social media addiction, Internet addiction, gaming disorder, ICD-11, SMDT, questionnaire, assessment, validation, psychometric properties, screening

## Abstract

While the use of video games and social media is an enjoyable recreational activity for most users, a minority develop problematic patterns of use. In the ICD-11, for the first time, there is a category of “disorders due to addictive behaviors” (including gaming disorder). The scientific literature also suggests a potential for the problematic use of social media. Economic screening tools for this are still needed. A very economical questionnaire to record gaming disorder is the ICD-11-based Gaming Disorder Test (GDT). The purpose of the present survey was to investigate the psychometric properties of an adapted questionnaire (Social Media Disorder Test, SMDT) to assess problematic social media use (PSMU). Therefore, 443 youths (mean age: 20.11 years) were examined with the SMDT and other measures regarding PSMU, self-control, and psychopathology. A confirmatory factor analysis (CFA) and reliability and correlation analyses were conducted. For the SMDT, we observed very good fit indices in the CFA, suggesting a one-factor structure; the reliability coefficient was 0.90, and we found the first indications for criterion validity. If the results can be confirmed, the SMDT, with its four questions, would be a very economical instrument to measure PSMU based on the ICD-11 criteria for behavioral addictions.

## 1. Introduction

The use of digital media is one of the most popular leisure activities. For example, according to current estimates worldwide, 4.62 billion people use social media [1], and more than 3 billion people play video games [2]. While the use of video games and social media is an enjoyable recreational activity for most users, a minority develop problematic patterns of use (e.g., [3]).

In an internationally established classification system, the problematic use of video games was incorporated as a research diagnosis (Internet gaming disorder, IGD) in the appendix of the DSM-5 for the first time [4]. The nine DSM-5 criteria for IGD are mental preoccupation, withdrawal symptoms, the development of tolerance, a loss of control, a loss of interest in other activities, continuation despite psychosocial consequences, the deception of others, dysfunctional coping, and risks or losses (all related to or caused by video games) [4]. These criteria must be checked for their occurrence in the previous 12 months, and at least five of the nine criteria must be met for a diagnosis of IGD [4]. In addition to the DSM-5, there are now also a number of psychometrically good screening instruments available (e.g., the Internet Gaming Disorder Scale–Short-Form or IGDS9-SF [5]) for recording these criteria.

For the first time, the current International Statistical Classification of Diseases and Related Health Problems of the World Health Organization (WHO), the ICD-11, contains a category termed “Disorders due to addictive behaviors”. This includes the problematic use of video games, classified as gaming disorder (GD), and gambling, classified as gambling disorder (e.g., [6]), based on very similar criteria. These criteria (for gaming) are (I) impaired control over gaming; (II) increasing priority given to gaming, to the extent that gaming takes precedence over other life interests and daily activities; and (III) the continuation or escalation of gaming despite the occurrence of negative consequences. Furthermore, the gaming behavior results in marked distress or a significant impairment in personal, family, social, educational, occupational, or other important areas of functioning [6]. For gambling disorder, the formulated ICD-11 criteria are almost identical. 

Even though it has not been labeled as a diagnosis in the ICD-11 thus far, a large body of the scientific literature also suggests a potential for the problematic use of social media (e.g., [7]). The first reviews show empirical links between a greater psychopathological burden and a poorer well-being and problematic social media use. A meta-analysis by Huang [8] included 133 independent samples and observed associations between problematic social media use and more pronounced depression and loneliness, as well as lower self-esteem and life satisfaction. In a systematic review, Lopes et al. [9] reported links with higher levels of depression and anxiety. As potential triggers for problematic social media use, deficits in self-control, e.g., [10], or emotion regulation, e.g., [11], are discussed.

Suitable diagnostic instruments are needed to conduct further research on both problematic behavioral patterns (problematic social media use and gaming/gambling disorder) and to identify affected individuals at an early stage, thus putting them within reach of preventive and interventive measures. Ideally, screening instruments should be economical in their implementation and evaluation, as well as precise in measuring the extent of problematic behavior patterns. The criteria of the ICD-11 provide a good basis for standardizing diagnostics. The ICD-11 criteria for gaming disorder are depicted very concisely in the Gaming Disorder Test (GDT) developed by Pontes et al. [12]. According to Cudo et al. [13], the GDT “…is one of the most widely utilised psychometric tools to assess GD symptoms according to the WHO framework” (p. 1). The GDT is currently available in various languages (e.g., Bengali, Chinese, English, German, Italian, Malay, Polish, Spanish, and Turkish). Despite its brevity (only four items with a five-level response format), it is characterized by good psychometric properties.

Regarding factor structure, Pontes et al. [12] first reported both the English-language version and a Chinese version of the GDT as single-factor structures. The one-dimensionality of the Chinese version of the GDT was confirmed by Wang and Cheng [14], as well as by Chen et al. [15] and Wu et al. [16]. Montag et al. [17] also observed a unidimensional factor structure for the German version of the GDT. For the Turkish version of the GDT, first Evren et al. [18], and afterwards Cakiroglu and Alnak [19], found a single-factor structure. Maldonado-Murciano et al. [20] obtained a one-factor structure for the Spanish GDT. Cudo et al. [13] reported a single-factor structure for the Polish version of the GDT. Islam et al. [21] presented a unidimensional factor structure for the Bangla GDT (after correlating the error variances of GDT items 2 and 3). Chiorri et al. [22] reported a single-factor structure for the Italian version of the GDT in the supplementary materials of their paper. Ghazi et al. [23] found a one-factor structure for the Malay language version of the GDT.

Regarding reliability, satisfactory to good reliability values were shown for the GDT. The values ranged between 0.78 [21] and 0.92 [13] for Cronbach’s alpha, as well as between 0.74 [22] and 0.93 [13] for McDonald’s omega. Empirical evidence of convergent and criterion-related validity (always concurrent validity up to now) were obtained in the investigations of Chen et al. [15], Chiorri et al. [22], Cudo et al. [13], Evren et al. [18], Ghazi et al. [23], Islam et al. [21], Maldonado-Murciano et al. [20], Pontes et al. [12], Wang and Cheng [14], and Wu et al. [16].

To sum up, there is clear empirical evidence for a single-factor structure of the GDT and a satisfactory to good reliability of the instrument. In addition, the available findings indicate its validity. The purpose of the present study is to investigate the psychometric properties of an adapted questionnaire (Social Media Disorder Test or SMDT) to assess problematic social media use. We examine whether the psychometric properties of the SMDT are comparable to those of the GDT (e.g., regarding the one-factor structure), which would make SMDT a very economical screening instrument to capture the problematic use of social media based on the ICD-11 criteria of the new category “Disorders due to addictive behaviors”. Moreover, we determine correlations with other characteristics (self-control and different aspects of mental health) to compare them with the already published findings on problematic social media use. Accordingly, we seek to answer the following research questions:

1. Does the newly developed SMDT also show a one-dimensional factor structure?
2. How reliable is the SMDT?
3. Can evidence be found to support the validity of the SMDT?

## 2. Materials and Methods

### 2.1. Procedure

The data used for this cross-sectional evaluation were collected between May and June 2023 as part of the seventh wave of the VEIF project (a longitudinal study) conducted by a market research institute. In this wave, the questions of the SMDT were asked for the first time. The Ethics Committee of the German Educational Research Association (GERA, German designation: Deutsche Gesellschaft für Erziehungswissenschaft, DGfE) approved this study (approval number: 01/2021/DGfE). Data were collected by 65 interviewers who visited the families at their homes. The VEIF project features a sample with an increased risk of problematic use of digital media compared to the general population. To recruit the sample, the oversampling of minors with an increased risk of problematic digital media use was carried out before the first data collection in 2016 (see Wartberg et al. [24] for a more detailed description of the survey design and the recruitment process conducted at the beginning of this research project).

### 2.2. Measures

We collected youths’ self-ratings of problematic social media use with the new Social Media Disorder Test (SMDT) by adapting the items of the GDT [12] to survey the problematic use of social media instead of video games in the previous 12 months. We used existing translation material for this process. The wording of the SMDT items remained very similar to the wording of the GDT items, but “gaming” was changed to “social media” in all four items (also, in both item 2 and item 3 of the English SMDT, we added the word “use” and adjusted the wording for item 3 in the German SMDT). The English version of the GDT only lists examples in parentheses for item 4 (see Pontes et al. [12], p. 524), while in the German translation of the GDT (introduced by Montag et al. [17]), examples are listed in brackets for item 3 and item 4. This approach was maintained for the SMDT. The examples previously used (both for item 3 and item 4) in the German GDT were minimally unified and supplemented in the German version of the SMDT (mainly regarding the situations of further education). Analogous to the GDT, the four items of the SMDT have a five-level response format (1 = “never”, 2 = “rarely”, 3 = “sometimes”, 4 = “often”, 5 = “very often”). The total score can be determined by summing up the answers to the four questions (range: 4 to 20). A higher score indicates a higher degree of self-assessed problematic social media use.

As an already established measure for problematic social media use within the last year, the Social Media Disorder Scale (SMDS, [25]) was additionally presented to the youths. The SMDS is based on adapted DSM-5 IGD criteria and is widely used internationally. The questionnaire consists of nine items with a binary response format (0 = “no”, 1 = “yes”). An example item from the SMDS is “During the past year, have you regularly neglected other activities (e.g., hobbies, sport) because you wanted to use social media?”. A sum value can be calculated for the nine questions, and a higher sum indicates more pronounced self-rated problematic social media use. The reliability coefficient of the SMDS in the sample at hand was 0.82.

Self-assessed problematic Internet use was explored with the widely used Young Diagnostic Questionnaire (YDQ) [26]. The YDQ comprises eight items with a binary response format (0 = “no”, 1 = “yes”). An example item from the YDQ is “Have you jeopardized or risked the loss of a significant relationship, job, educational, or career opportunity because of the Internet?”. A total score can be calculated by summing up the eight answers. A higher sum indicates a higher degree of problematic Internet use. The reliability coefficient of the YDQ in the surveyed sample was 0.77.

Self-rated self-control was examined with a short form of the Self-Control Scale (SCS, [27]) called SCS-K-D [28]. The SCS-K-D consists of 13 items with a five-level response format (from 1 = “completely incorrect” to 5 = “completely accurate”). A mean value is calculated from all questions. An example item from the SCS-K-D is “Sometimes I cannot stop myself from doing something, even though I know it is wrong.”. A higher mean indicates a higher degree of self-assessed self-control. The reliability (Cronbach’s alpha) of the SCS-K-D in the sample investigated was 0.85.

The self-assessment of the youths’ mental health in the previous six months was conducted with the German adaption of the Reynolds Adolescent Adjustment Screening Inventory [29]: Screening psychischer Störungen im Jugendalter-II (SPS-J-II, [30]). The SPS-J-II comprises 32 items with a three-level response format (0 = “never or almost never”, 1 = “sometimes”, 2 = “nearly all the time”). Based on the answers to the 32 questions, four subscales (“antisocial behavior”, “anger control problems”, “emotional distress” (a combined measure of anxiety and depressiveness), and “self-esteem problems”) can be calculated. An example item from the subscale antisocial behavior is “In the last six months, I have done things that were against the law.”. An example item from the subscale anger control problems is “In the last six months, I have lost my temper.”. An example item from the subscale emotional distress is “In the last six months, I have been very worried about the future.”. An example item from the subscale self-esteem problems is “In the last six months, I found that everything in my life was okay.”. In each scale, a higher sum value indicates a more pronounced self-rated psychopathological burden. The reliability coefficients (Cronbach’s alpha) in the sample examined were as follows: (I) antisocial behavior: 0.80, (II) anger control problems: 0.82, (III) emotional distress: 0.88, and (IV) self-esteem problems: 0.77. Finally, socio-demographic characteristics (e.g., gender and the ages of the youth and their parents), as well as the social media usage times of the youth, were collected.

### 2.3. Sample Description

The sample consisted of 443 youths (aged between 19 and 22 years, with 45.1% of the youth or 200 being female persons and 54.9% or 243 being male persons) and 446 related caregivers. Overall, 88.3% of the caregivers were biological mothers and 11.1% biological fathers of the youth, while 0.7% were new partners of the mothers or stepfathers (in the following, all of them are called “parents”). The mean age of the youth was 20.11 years (SD = 0.91), and that of their parents was 47.72 years (SD = 4.57). Most of the youth (87.8% or 389 cases) still lived in the same household as their parent, while 2.5% reported still having a room in the household of their parents, in addition to their own apartment. A percentage of 3.8% lived alone in an apartment, and another 5.9% shared an apartment with others. A total of 82.4% of the young people (365 cases) had already finished school (8.8% of them had graduated from school at a low education level, 56.7% at a medium education level, 34.2% at a high education level, and 0.3% had left school without graduation). The parents of the remaining youth still attending school estimated that 6.3% will graduate at a low education level, 20.0% at a medium education level, and 73.8% at a high education level.

### 2.4. Statistical Analyses

A total of 446 external assessments by parents and 443 self-ratings by youths were collected. Here, only the data from the 443 youths were used for further statistical analyses. For the sample description, means and standard deviations, as well as frequencies, were calculated. To explore the psychometric properties of the SMDT, independent samples *t*-tests, a confirmatory factor analysis or CFA (estimation procedure: maximum likelihood or ML) and reliability and correlation analyses were conducted. According to the published findings for the GDT [12,13,14,15,16,17,18,19,20,21,22,23], we postulated in the CFA a unidimensional structure, with the four items of the SMDT loading on a single latent factor. The normed *χ*^2^ index, the Root Mean Square Error of Approximation (RMSEA), the Standardized Root Mean Square Residual (SRMR), the Comparative Fit Index (CFI), and the Tucker–Lewis Index (TLI) were used to assess the global goodness-of-fit of the CFA model. Additionally, as local parameters of model fit, standardized factor loadings were also explored in the CFA. Most calculations were performed with the statistical software SPSS (version 27.0, IBM, 2020, New York, NY, USA), but the CFA was conducted with the software Mplus (version 8.10 [31]).

## 3. Results

### 3.1. Descriptive Statistics for the SMDT

In the sample of the present survey, the mean value of the SMDT was 5.78 (SD = 2.73, range: 4 to 20). For the independent samples *t*-tests, the group was divided by gender, and no statistically significant difference (t = −1.73, df = 440.53, *p* = 0.084) was observed in the SMDT sum value between the self-ratings of the female (M = 5.54, SD = 2.37) and male youths (M = 5.98, SD = 2.98). For the total value of the SMDS, there was also no difference (t = −0.80, df = 422.69, *p* = 0.426) between the women and men.

### 3.2. Factor Structure of the SMDT

Regarding the results of the CFA, we observed the local parameters of the model fit (standardized factor loadings) for the four SMDT items to be between 0.80 and 0.87 (see Table 1 for the wording of the items and the loadings). The normed χ^2^ index (χ^2^/df) for the SMDT was 0.188 (χ^2^ = 0.375, df = 2, *p* = 0.829). For the global goodness-of-fit of the model, we found values of 0.000 for the RMSEA (90% Confidence Interval: 0.000 to 0.055) and 0.002 for the SRMR, as well as 1.000 each for the CFI and the TLI. The empirically determined values for the RMSEA, the SRMR, the CFI, and the TLI clearly reached the cut-off values for a good model fit recommended by Schermelleh-Engel et al. [32] (RMSEA ≤ 0.05, SRMR ≤ 0.05, CFI ≥ 0.97, and TLI ≥ 0.97). Accordingly, the observed values in the CFA clearly seem to indicate a one-dimensional structure of the SMDT.

### 3.3. Reliability of the SMDT

We obtained a reliability coefficient (Cronbach’s alpha) of 0.90. According to the common standards, in the sample investigated, the internal consistency of the SMDT can be considered good (e.g., [33]).

### 3.4. Criterion Validity of the SMDT

We found statistically significant correlation coefficients between the SMDT and the Social Media Disorder Scale (SMDS, r = 0.58, *p* < 0.001), as well as the weekly social media usage time (r = 0.21, *p* < 0.001). These findings can be interpreted as the first indications of criterion validity for the SMDT (see Table 2).

## 4. Discussion

The aim of the present study was to examine the psychometric properties of a new instrument (Social Media Disorder Test, SMDT) for measuring problematic social media use. The SMDT, for examining problematic social media use, represents an adaptation of the Gaming Disorder Test (GDT), which, according to Cudo et al. [13], is one of the most frequently used instruments to investigate the ICD-11 diagnosis of gaming disorder. The SMDT is also based on the ICD-11 criteria catalog of the WHO for disorders due to addictive behaviors (although there is no diagnosis for problematic social media use so far). In answer to the first research question, the results of the conducted CFA clearly indicate a one-dimensional factor structure for the SMDT. In response to the second research question, good reliability (α = 0.90) was obtained for the SMDT. In response to the third research question, initial evidence of criterion validity was observed for the SMDT (see Table 2). In the correlation analyses for the SMDT, according to the proposal of Cohen [34], a large effect size (Pearson’s product–moment correlation coefficient r > 0.50) was shown for the association with a DSM-5-based and internationally widely used measure for problematic social media use (Social Media Disorder Scale or SMDS) and a small effect size (r > 0.10) for the relationship with weekly social media usage time. Some researchers describe problematic Internet use as an overarching construct for problematic social media use, e.g., [25], and a moderate correlation (r > 0.30) between the two constructs was obtained in the present survey (see Table 2).

In addition, other correlations emerged in the present investigation that fit well with previously published findings on problematic social media use (these empirical results can also be found in Table 2). In analogy with the statistically significant association in the present study, Leijse et al. [10] also reported an association between problematic social media use and lower self-control. Previously, Wartberg and Kammerl [35] reported associations between problematic social media use and higher levels of antisocial behavior, as well as anger control problems (corresponding with the findings of the present survey). Furthermore, in their systematic review and meta-analysis, Shannon et al. [36] observed relationships between problematic social media use and increased anxiety and depression in adolescents and young adults, which fits in well with our finding on emotional distress (in Table 2). Also, in line with a result of the present study, Schivinski et al. [37] found a relationship between problematic social media use and lower self-esteem. In summary, we found links between psychological characteristics and the new SMDT instrument (see Table 2) that are very comparable to empirical findings based on other questionnaires for measuring the problematic use of social media.

This survey has several limitations. In the present study, no representative sample was investigated. Matching the SMDT with the results of a standardized structured interview (such as that developed by Koo et al. [38] for the DSM-5 research diagnosis Internet gaming disorder) would be desirable; however, the authors are not aware of any such interview for problematic social media use. These are the very first psychometric findings for the new SMDT instrument that was tested in a sample of only young persons. In this survey, people in a very narrow age range (19 to 22 years) were examined with the SMDT, and the transferability of the reported findings to other age groups cannot be taken for granted. The psychometric properties of the SMDT should be checked again in further studies (e.g., for people in middle and older adulthood, as well as for children and younger adolescents). Verifications of certain types of validity (e.g., regarding predictive validity) are not possible due to the cross-sectional design. Additional checks of convergent validity with other established instruments for problematic social media use (e.g., the Bergen Social Media Addiction Scale [39]) would be informative in future studies. To summarize, the SMDT requires further testing in other age groups, translations into other languages are needed (we applied the represented German SMDT, but an English version of the SMDT is also suggested in Table 1), and additional psychometric aspects should be tested before its extensive suitability can be assessed.

## 5. Conclusions

This initial test indicates good psychometric properties of the SMDT. The SMDT was derived from the GDT, and the properties of both instruments seem to be very similar (see, e.g., [22] for the GDT). If these findings (one-dimensionality, good reliability, and validity) are confirmed in other samples, the SMDT would be a very economical screening instrument for problematic social media use. As far as we know, there is no instrument that measures problematic social media use with only four items. Other screening questionnaires on this behavioral pattern are somewhat longer. For instance, the DSM-5-based Social Media Disorder Scale (SMDS) comprises 9 questions in a shortened form instead of 27 items [25]. If problematic social media use is to be examined in scientific studies but only very few questions can be utilized (for example, in large population samples in which many different subject areas are to be covered), the SMDT would be a reliable and valid option. Another advantage of the newly presented SMDT is that it transfers the diagnostic criteria defined in the ICD-11 for gaming disorder and gambling disorder (which are very similar) to a further problematic behavior pattern in the subject area of behavioral addictions and could thus contribute to the standardization of diagnostics according to the ICD-11.

## Figures and Tables

**Table 1:** Items and factor loadings for the Social Media Disorder Test.

Item Number	Wording of SMDT	German Wording of SMDT	Factor Loadings
	Please indicate how often the following issues occurred on average over the past twelve months until today.	Bitte gib an, wie häufig die folgenden Probleme im Durchschnitt über die letzten zwölf Monate hinweg bis zum heutigen Tage aufgetreten sind.	
Item 1	I have had difficulties controlling my social media activity.	Ich habe Probleme gehabt, meine Social Media-Aktivitäten zu kontrollieren.	0.80
Item 2	I have given increasing priority to social media use over other life interests and daily activities.	Ich habe Social Media steigende Priorität gegenüber anderen Lebensinteressen und täglichen Aktivitäten eingeräumt.	0.81
Item 3	I have continued social media use despite the occurrence of negative consequences.	Ich habe Social Media weiterhin genutzt, obwohl negative Konsequenzen entstanden sind (z.B. in Beziehungen, Schule, Ausbildung, Studium oder Job).	0.87
Item 4	I have experienced significant problems in life (e.g., personal, family, social, education, occupational) due to the severity of my social media behavior.	Ich habe bedeutsame Probleme in meinem Leben aufgrund der Stärke meines Social Media-Verhaltens erfahren (z.B. persönlich, familiär, sozial, in der Schule, der Ausbildung, dem Studium oder im Beruf).	0.86

**Table 2:** Correlation matrix for the SMDT and the other psychosocial aspects.

Variable	1	2	3	4	5	6	7	8	9
(1) Social Media Disorder Test ^a^	–								
(2) Social Media Disorder Scale ^b^	0.58 ***	–							
(3) Weekly social media usage time	0.21 ***	0.28 ***	–						
(4) Problematic Internet use	0.44 ***	0.67 ***	0.12 *	–					
(5) Self-control	−0.45 ***	−0.33 ***	−0.14 **	−0.33 ***	–				
(6) Antisocial behavior	0.60 ***	0.21 ***	0.06	0.13 **	−0.45 ***	–			
(7) Anger control problems	0.57 ***	0.26 ***	0.12 *	0.18 ***	−0.56 ***	0.79 ***	–		
(8) Emotional distress	0.55 ***	0.27 ***	0.07	0.23 ***	−0.45 ***	0.64 ***	0.62 ***	–	
(9) Self-esteem problems	0.48 ***	0.30 ***	0.04	0.22 ***	−0.49 ***	0.45 ***	0.55 ***	0.54 ***	–

Note. ^a^ = SMDT; ^b^ = SMDS; *** *p* < 0.001; ** *p* < 0.01; * *p* < 0.05.

## Data Availability

The data presented in this study are available on reasonable request from the corresponding author.
